# Can Molecular Docking Predict Guest Orientation in β-Cyclodextrin? A Cross-Docking and DFT Refinement Study

**DOI:** 10.3390/molecules31193379

**Published:** 2026-09-23

**Authors:** Ewa Napiórkowska, Konrad Adam Michalik, Łukasz Szeleszczuk

**Affiliations:** 1 Department of Organic and Physical Chemistry, Faculty of Pharmacy, Medical University of Warsaw, Banacha 1 Str., 02-093 Warsaw, Poland; ewa.napiorkowska@wum.edu.pl (E.N.); konrad.michalik@student.wum.edu.pl (K.A.M.); 2 Doctoral School, Medical University of Warsaw, Żwirki i Wigury 81 Str., 02-093 Warsaw, Poland

**Keywords:** β-cyclodextrins, molecular docking, guest properties, DFT, inclusion complexes

## Abstract

Molecular docking is widely used to predict cyclodextrin host–guest structures, yet its ability to reproduce the directional orientation of asymmetric guests within the β-cyclodextrin (β-CD) cavity has rarely been evaluated systematically. Here, 20 crystallographically characterized 1:1 β-CD complexes containing asymmetric 1,4-disubstituted benzene guests were selected from the Cambridge Structural Database. Each guest was docked into each of 20 experimentally derived β-CD conformations, yielding 7916 retained poses. Poses reproducing the crystallographic direction of inclusion were classified as Up, whereas the opposite orientation was classified as Down. Across the complete dataset, approximately 26% of poses were Up, with guest-dependent frequencies ranging from 5% to 83%. Importantly, only one β-CD conformation generated Up poses for all 20 guests, whereas the complete receptor set sampled at least one crystal-consistent pose for every compound. However, GlideScore-based orientation recovery was 65% for Top-1, 95% for Top-2, and 100% for Top-3, demonstrating that successful sampling does not necessarily ensure correct top-ranked prediction. Selected poses were subsequently optimized using dispersion-corrected DFT. DFT-based energetic ranking did not consistently favour the crystallographic orientation and could either decrease or increase guest heavy-atom RMSD. Overall, the results demonstrate that reliable validation of β-CD docking requires explicit separation of pose generation, pose ranking, energetic refinement, and structural similarity, and support the use of structurally diverse crystal-derived receptor sets to broaden orientational sampling.

## 1. Introduction

Cyclodextrins (CDs) are cyclic oligosaccharides composed of α-D-glucopyranose units connected by α-1,4-glycosidic bonds. The three most common native cyclodextrins, α-, β-, and γ-CD, contain six, seven, and eight glucopyranose units, respectively [1,2,3]. Their three-dimensional structure is commonly described as a truncated cone, with primary hydroxyl groups forming the narrower rim and secondary hydroxyl groups forming the wider rim. The external surface is relatively hydrophilic, whereas the internal cavity is less polar and can accommodate molecular fragments of appropriate size [1,2,3]. Atomistic studies have nevertheless shown that this simplified representation does not fully capture the conformational dynamics and hydration of the CD cavity, both of which can contribute substantially to molecular recognition [4,5].

The ability of CDs to form reversible non-covalent inclusion complexes underlies their broad range of applications. Complexation can improve aqueous solubility, protect chemically or photochemically labile compounds, reduce volatility, modify dissolution and release, and enhance the apparent bioavailability of poorly soluble molecules [2,3,6,7,8]. Consequently, CDs are widely employed in pharmaceutical formulation, food technology, analytical and separation sciences, and supramolecular materials [6,7,8,9,10,11]. Among native cyclodextrins, β-CD is particularly important because its seven glucopyranose units provide a cavity suitable for a broad range of aromatic and moderately sized hydrophobic guests. Its availability, extensive physicochemical characterization, and large body of structural data have made it one of the most frequently investigated hosts in pharmaceutical and supramolecular chemistry [1,2,7]. β-CD is also widely used as a chiral selector, illustrating the sensitivity of its molecular recognition to relatively subtle differences in guest geometry and intermolecular interactions [12].

Formation of a β-CD inclusion complex is not governed by size complementarity alone. Host–guest association reflects a balance of dispersion and van der Waals interactions, hydrogen bonding, electrostatic effects, conformational strain, desolvation, and changes in the organization of water associated with the host cavity and guest [4,5,13]. An additional structural feature is the intrinsic directional asymmetry of β-CD. Its primary and secondary rims differ geometrically and chemically, creating two nonequivalent environments at the entrances to the cavity. Consequently, an asymmetric guest can often be accommodated in more than one direction, with different molecular fragments exposed toward the narrow and wide rims. Experimental and computational studies have demonstrated that such inclusion geometries depend sensitively on guest structure, host conformation, environment, and intermolecular interactions [14,15,16,17,18].

A variety of experimental methods, including X-ray diffraction, NMR spectroscopy, calorimetry, thermal analysis, and spectroscopic techniques, are used to characterize CD inclusion complexes [6,13,14,15,16,17,18]. Computational chemistry provides complementary molecular-level information that is often difficult to obtain experimentally. Molecular dynamics simulations can describe host flexibility, guest mobility, solvent organization, and alternative binding modes [19], whereas quantum-chemical calculations can provide detailed information on optimized structures, energetics, hydrogen bonding, dispersion interactions, and electronic properties [20]. Combined experimental and computational approaches have therefore become increasingly important in studies of CD host–guest systems [15,16,17,18,19,20].

Molecular docking occupies a particularly useful position within this computational framework because it can rapidly generate and rank large numbers of plausible host–guest arrangements at substantially lower computational cost than extensive molecular dynamics simulations or quantum-chemical optimization. In cyclodextrin studies, docking is commonly used to propose inclusion modes, predict the direction of guest insertion, identify possible intermolecular contacts, and generate starting structures for subsequent MD or DFT calculations [21,22,23,24]. Its application to CD-based supramolecular systems has been reviewed extensively, and numerous recent studies demonstrate its practical value for proposing structures of β-CD complexes [21,22,23,24].

Nevertheless, docking to cyclodextrins presents methodological challenges that differ from those encountered in many conventional protein–ligand systems. β-CD is comparatively small and conformationally flexible, and changes in glycosidic torsions or hydroxyl-group orientations can modify both cavity geometry and the distribution of interaction sites. Moreover, the cavity is accessible through two nonequivalent openings. An asymmetric guest may therefore adopt two opposite inclusion modes that retain similar overall cavity occupation while reversing the positions of its substituents relative to the wide and narrow rims [15,16,24,25]. Both arrangements may preserve favorable inclusion of an aromatic core and establish alternative interactions at the cavity entrances.

This situation creates separate challenges for the sampling and scoring stages of docking. A docking algorithm may successfully generate the experimentally observed orientation but fail to rank it as the most favorable pose. Conversely, a favorable docking score does not establish that the predicted structure reproduces the experimental direction of inclusion. Docking scores are ranking functions and should not be interpreted directly as physical binding free energies. More sophisticated calculations of cyclodextrin host–guest affinities likewise remain sensitive to conformational sampling, energetic models, solvent treatment, and entropy [26]. Thus, the ability to generate a crystal-consistent pose and the ability to identify that pose as preferred are distinct aspects of docking performance.

Host flexibility further complicates this problem. β-CD does not possess a unique host geometry, even among experimentally determined structures. A recent large-scale analysis identified 437 β-CD conformations extracted from 293 Cambridge Structural Database entries and revealed substantial structural and energetic diversity among them [27]. Host selection therefore cannot necessarily be treated as a neutral preprocessing step. A β-CD conformation appropriate for one guest may be less suitable for another, providing a direct rationale for ensemble docking, in which multiple host conformations are evaluated rather than relying on a single arbitrarily selected structure.

Experimentally determined crystal structures offer a particularly valuable basis for validating such approaches. The Cambridge Structural Database (CSD) provides a curated collection of experimental structures that can serve as both a source of β-CD conformations and a reference for inclusion geometries [28]. However, crystallographic structures reflect not only direct host–guest interactions but also crystallographic water, neighboring molecules, intermolecular hydrogen bonding, and crystal packing. Consequently, removal of the crystal environment followed by computational optimization may lead to a different local structure without invalidating the experimentally determined geometry [16,17,18,27]. Importantly, β-CD geometries extracted from different crystal structures should not be interpreted as samples from a thermodynamic equilibrium conformational ensemble. Each structure reflects a particular crystalline environment and may be influenced by guest identity, hydration, crystal packing, intermolecular hydrogen bonding, and other solid-state interactions. In the present study, the CSD-derived β-CD structures are therefore used as a structurally diverse receptor set for probing the sensitivity of docking outcomes to host geometry, rather than as an approximation of the equilibrium conformational distribution of β-CD.

Despite the widespread use of docking for individual cyclodextrin complexes, systematic evaluation of its ability to recover the directional orientation of asymmetric β-CD guests remains comparatively limited [21]. Conventional validation based primarily on docking score or guest root-mean-square deviation (RMSD) does not fully address this problem. A guest may reproduce the experimental direction of inclusion while differing in insertion depth, tilt, or lateral displacement and therefore exhibit a relatively high RMSD. Conversely, a pose may occupy a similar region of the cavity while being directionally inverted. Directional agreement and atom-by-atom geometrical similarity should therefore be considered complementary validation criteria.

Guests containing a 1,4-disubstituted benzene ring with two different substituents provide a convenient model for examining this problem. Their common aromatic core is compatible with β-CD inclusion, whereas the nonequivalent para substituents allow reversal of the guest to be identified unambiguously. The resulting system can therefore test whether docking recognizes the directional asymmetry of β-CD rather than merely favourable accommodation of an aromatic group within the cavity.

Accordingly, the aim of the present study was to systematically evaluate the ability of molecular docking to reproduce the experimentally observed directional orientation of asymmetric guests within β-cyclodextrin. Twenty crystallographically characterized 1:1 β-CD inclusion complexes deposited in the CSD were selected as an experimental reference set. Each guest contained a 1,4-disubstituted benzene moiety containing two different substituents and was docked into each of the 20 experimentally derived β-CD conformations, enabling both redocking and cross-docking analyses. The resulting poses were classified as crystal-consistent (Up) or directionally inverted (Down) according to the orientation of the guest relative to the wide and narrow β-CD rims. Pose frequencies and GlideScore (G-Score) values were analyzed separately to distinguish the ability of the docking procedure to generate the experimental orientation from the ability of the scoring function to rank it correctly. Selected Up and Down complexes were subsequently subjected to dispersion-corrected DFT geometry optimization and thermochemical analysis, while guest heavy-atom RMSD after host-based alignment was used as a complementary measure of detailed structural agreement. This workflow was designed to clarify the respective roles of host conformation, orientational sampling, scoring, and higher-level structural refinement in the computational prediction of β-CD inclusion complexes.

For clarity, a simplified two-dimensional schematic representation of the host–guest systems investigated in this study is shown in Figure 1. The scheme illustrates the β-cyclodextrin host, the general asymmetric 1,4-disubstituted benzene guest motif used in the benchmark, and the definition of the Up and Down inclusion orientations relative to the wider and narrower rims of β-CD.

## 2. Results and Discussion

### 2.1. Docking-Score Variability Across the Guest and β-CD Conformational Sets

The ability of molecular docking to reproduce the experimentally observed direction of guest inclusion was evaluated using asymmetric 1,4-disubstituted benzene derivatives for which the two ends of the molecule could be distinguished unambiguously (Table S1). A pose reproducing the direction of inclusion observed in the corresponding crystal structure is referred to throughout this study as Up, whereas the directionally inverted pose is denoted Down. This classification deliberately focuses on the orientation of the guest relative to the wide and narrow rims of β-CD and does not require exact reproduction of smaller differences in insertion depth, inclination, or lateral displacement within the cavity.

The variability of GlideScore values obtained for each guest across the complete set of β-CD conformations is summarized in Table 1. For most compounds, the difference between the least and most favorable docking scores was relatively moderate, generally amounting to approximately 0.9–2.0 score units. The narrowest range was observed for KUTJUE_G (0.94), followed by QACXEX_G (1.08) and UPULEX_G (1.30). In contrast, AJUVAC_G displayed a markedly broader range of 5.24, with GlideScore values extending from −0.22 to −5.46, while XADHIT_G showed the second-largest range, 3.27, from −2.78 to −6.04. Thus, the score assigned to some guests was comparatively insensitive to host selection, whereas other guests showed a pronounced dependence on the particular β-CD conformation used for docking.

Several structurally related guests showed qualitatively similar GlideScore profiles. FODBEG_G and FODBIK_G, for example, displayed closely comparable maximum and minimum values and score ranges of 1.65 and 1.49, respectively. KOFJEU_G and KUTJUE_G also exhibited relatively similar score intervals, whereas OFAXID_G and QACXEX_G showed closely related most favorable GlideScore values. These observations are consistent with an influence of common guest structural features on the response of the scoring function. However, the present dataset is not intended to establish a quantitative structure–score relationship, and similarity in the overall GlideScore range did not necessarily translate into similar orientational behaviour.

This distinction is particularly important for β-CD because successful placement of the aromatic portion of the guest within the cavity does not automatically imply successful reproduction of the crystallographic direction of inclusion. Owing to the directional asymmetry of the host, two poses can occupy a similar region of the cavity while exposing different substituents toward the wide and narrow rims. Consequently, docking-score magnitude and directional correctness represent related but non-equivalent aspects of docking performance.

### 2.2. The Frequency of Crystal-Consistent Orientations Depends Strongly on Both Guest Identity and β-CD Conformation

The orientation of all 7916 retained docking poses was classified as Up or Down. The corresponding percentages for every guest–β-CD combination are summarized in Table 2. Across the complete dataset, approximately 26% of the retained poses were classified as Up. Because the retained poses are clustered within guest–receptor docking calculations, this value is reported only as a descriptive pose-level statistic and should not be interpreted as arising from 7916 independent observations. At the guest level, the mean proportion of Up poses was 26.0%, with a median of 18.4% and an interquartile range of 13.4–31.0%. The corresponding guest-level bootstrap 95% confidence interval for the mean was 17.7–36.0%. Thus, the docking procedure was capable of generating crystal-consistent orientations, but the overall ensemble of retained solutions was dominated by directionally inverted poses.

The frequency of Up poses differed markedly among individual guests. LACZEW_G showed the lowest overall proportion, with only 5% of its retained complexes classified as Up. FEXBOA_G and HAKPUG_G similarly displayed a pronounced preference for the Down class, with only 8% Up poses. At the opposite extreme, UPULEX_G produced 83% Up complexes and XADHIT_G 80%. Intermediate behaviour was observed for several other guests; for example, KOFJEU_G, KUTJUE_G, and CIQFIU_G generated approximately 40–42% crystal-consistent poses.

The representative examples collected in Table 3 illustrate the magnitude of this variation. FEXBOA_G produced only 32 Up poses among 400 retained complexes, whereas XADHIT_G produced 320. KUTJUE_G and CIQFIU_G occupied an intermediate region, with 154/400 and 167/400 Up poses, respectively. The strong variation between chemically different guests demonstrates that their intrinsic structural characteristics contribute substantially to the probability of generating the crystallographic orientation.

Guest identity alone, however, did not determine the docking outcome. For most compounds, changing only the β-CD conformation produced pronounced changes in the Up/Down distribution. For an individual guest, the percentage of Up poses could vary from zero for one host conformation to well above 50%, and in several cases approached or reached 100% for another. Therefore, a guest that was rarely placed in the crystallographic direction by one experimentally derived β-CD conformation could be oriented efficiently by another.

A similarly strong dependence was observed from the host perspective. Averaged over the complete guest set, individual β-CD conformations generated between approximately 15 and 54% Up complexes. LACZEW_CD and CIQFIU_CD showed the highest overall percentages, 54 and 51%, respectively, whereas several other host conformations generated only approximately 15–17% Up poses. Experimentally observed β-CD conformations therefore cannot be regarded as equivalent docking receptors.

The ability of a particular β-CD conformation to generate Up poses also did not follow the relative Gibbs-energy ordering used to arrange the host structures in Table 2. The proportion of crystal-consistent poses showed no monotonic trend along this ordering. Notably, LACZEW_CD, located toward the higher-energy end of the conformational set, generated the largest overall proportion of Up complexes, whereas several lower-energy conformations performed considerably less favorably.

Consequently, intrinsic energetic preference of a β-CD conformer does not provide a straightforward criterion for selecting a universally optimal host for docking. A host conformation that is relatively unfavorable in the isolated conformational ranking may nevertheless possess a cavity geometry that is particularly suitable for generating the crystal-consistent orientation of a particular guest.

The most important sampling result emerged when the complete crystal-derived receptor set was considered. Among all tested β-CD structures, only LACZEW_CD generated Up complexes for all investigated guests. In contrast, the complete set of 20 CSD-derived β-CD receptor structures produced at least one Up pose for every guest. Thus, the principal benefit of using a structurally diverse receptor set in the present benchmark was increased coverage of orientational space. This observation should not be interpreted as implying that the receptor set represents an equilibrium β-CD conformational ensemble. Rather, structural diversity among the crystal-derived hosts increased the probability that the experimentally observed orientation was represented somewhere among the generated solutions.

This distinction is important because ensemble docking should not be interpreted as guaranteeing prediction of the crystallographic orientation. Rather, host conformational diversity increased the probability that the experimentally observed orientation was represented somewhere among the generated solutions, thereby addressing the sampling component of the docking problem.

FEXBOA_G provides a clear visual illustration of this behaviour (Figure 2). Superposition of the corresponding poses reveals two distinguishable directional populations within the β-CD cavity. Both Up and Down orientations were sampled, but only 8% of the retained FEXBOA_G complexes reproduced the crystallographic orientation. The two pose classes therefore represent a genuine reversal of guest direction relative to the nonequivalent β-CD rims rather than mere differences in penetration depth or inclination. This finding supports the use of Up/Down classification as a complementary structural criterion in the validation of cyclodextrin docking.

### 2.3. Sampling and Prediction of the Crystal-Consistent Orientation Are Distinct Outcomes

The presence of an Up pose among the retained docking solutions demonstrates that the experimentally observed orientation can be sampled, but does not demonstrate that it can be predicted from the docking results. We therefore distinguished explicitly between orientation sampling and ranking-based orientation recovery. Sampling was considered successful when at least one crystal-consistent Up pose was generated for a given guest across the complete set of crystal-derived β-CD receptors. To evaluate ranking performance, all retained poses generated for each guest across the 20 receptor conformations were ranked according to GlideScore, and Top-N recovery was considered successful when at least one Up pose was present among the N highest-ranked poses (Table S2 in Supplementary Materials).

The complete host set sampled at least one Up pose for all 20 investigated guests, corresponding to a sampling success of 100%. In contrast, the globally best-scoring pose was Up for 13 of 20 guests, corresponding to a Top-1 orientation-recovery rate of 65.0% [95% CI: 43.3–81.9%]. Recovery increased to 95.0% (19/20) for Top-2 and 100% (20/20) for Top-3; Top-5 recovery was likewise 100% (see Table S3 in Supplementary Materials).

As a simple reference baseline, assuming randomly assigned Up and Down orientations with equal probability, the expected probability of recovering at least one Up pose is 50.0% for Top-1, 75.0% for Top-2, 87.5% for Top-3, and 96.9% for Top-5. The rapid increase in this baseline with N illustrates that Top-N recovery becomes progressively less stringent in a binary orientation problem. Accordingly, Top-1 recovery provides the most demanding measure of ranking-based predictive performance, whereas Top-2 and Top-3 provide complementary information on whether the experimental orientation remains among the highest-ranked alternatives.

Importantly, several Up/Down comparisons were nearly degenerate according to the scoring function. The difference between the global Up and Down minima was only approximately 0.01 score unit for KUTJUE_G and LILLUN_G. Small differences were also observed for VOQDUA_G, KOFJEU_G, XUXFAY_G, HAKPUG_G, and UPULEX_G. Such differences provide little practical basis for assigning one of two directionally opposite poses as uniquely preferred (Table 4).

The docking score alone may therefore be insufficient to identify the crystallographic orientation even when that orientation has been sampled successfully. This limitation is particularly relevant in a genuinely predictive application, in which the experimental structure is not available, and two oppositely oriented poses with very similar scores cannot be classified retrospectively.

The comparison of Table 2 and Table 4 additionally demonstrates that pose frequency and score ranking are distinct descriptors of docking performance. XADHIT_G provides a particularly striking example. Approximately 80% of its retained docking poses were classified as Up, demonstrating that the crystal-consistent orientation was sampled readily. Nevertheless, the globally best Down pose obtained a GlideScore of −6.04, compared with −5.52 for the globally best Up pose. Thus, the orientation generated most frequently was not the orientation favored by the scoring function.

The converse situation is also possible: a relatively small Up population can contain an individual pose with a highly competitive or globally favorable GlideScore. Consequently, validation based only on the number of generated crystal-consistent poses or only on the top-ranked pose provides an incomplete description of docking behaviour.

Redocking to the β-CD conformation extracted from the corresponding crystal structure did not eliminate the sampling problem. No retained REDOCK_Up pose was obtained for HAKPUG_G, KOFJEU_G, NILQUV_G, or XUXFAY_G, whereas XADHIT_G did not yield a retained REDOCK_Down pose. In contrast, an Up orientation was obtained for every guest when the complete set of crystal-derived β-CD hosts was considered. This difference provides direct evidence that the experimentally matched host conformation does not necessarily sample both directional alternatives effectively.

The identity of the host conformation generating the globally best pose also varied strongly among guests. No single β-CD conformation consistently generated MIN_Up structures across the dataset. Moreover, only three of the 40 orientation-specific global GlideScore minima originated from the β-CD structure belonging to the corresponding crystallographic complex: LILLUN_G and LACZEW_G for the Up orientation, and CIQFIU_G for the Down orientation.

Thus, the use of the experimentally matched host structure does not guarantee the most favorable empirical docking result. This observation is relevant to the interpretation of conventional redocking experiments, which represent a relatively favorable validation scenario because the host has already adopted a geometry associated with the crystallographic guest. Nevertheless, the isolated host used for docking no longer experiences the complete crystallographic environment. Crystal packing, intermolecular contacts, and crystallographic water molecules can all contribute to the experimentally observed β-CD geometry, whereas these effects are absent from the docking models used here.

### 2.4. DFT Refinement Provides Complementary, Model-Dependent Energetic Information

The occurrence of oppositely oriented poses with similar GlideScore values motivated a second level of analysis based on quantum-chemical geometry optimization. The systematically selected MIN_Up, MIN_Down, REDOCK_Up, and REDOCK_Down complexes, together with the corresponding crystal-derived reference structures, were geometry-optimized using B3LYP/6-31G(d,p) with Grimme D3 dispersion correction. No implicit solvent model or explicit solvent molecules were included in the DFT calculations. The purpose of these calculations was comparative structural and energetic refinement of selected docking-derived geometries rather than quantitative prediction of host–guest binding free energies. Accordingly, the resulting energetic differences should be interpreted within the limitations of the applied gas-phase DFT model and basis set.

For thermochemical comparison, the Gaussian quantity corresponding to the sum of the electronic and thermal free energies was used. For each guest, the crystal-derived structure optimized under the same computational conditions was taken as the reference, and the relative value ΔG was defined as:

(1) $$\Delta G=G_{optimized\_docking-derived}-G_{optimized\_crystal-derived}$$

The resulting values are therefore relative gas-phase thermochemical estimates for optimized host–guest structures of identical composition. They must not be interpreted as binding free energies because no thermodynamic cycle involving separately calculated guest free energies was used. In particular, basis-set incompleteness and the absence of solvent polarization and explicit host–solvent/guest–solvent interactions may influence the relative ordering of alternative Up and Down structures, especially when their calculated energy differences are small.

The directional classification of the complexes remained unchanged during unconstrained geometry optimization: no structure initially classified as Up became Down, and no Down structure became Up. Geometry refinement therefore altered the detailed host–guest arrangement without reversing the overall direction of inclusion.

The DFT analysis showed that poses that were nearly indistinguishable by GlideScore could become substantially separated in calculated G after geometry relaxation. The FEXBOA_G–AJUVAC_CD system shown in Figure 3 provides a particularly clear example. Before DFT optimization, the Up and Down poses had GlideScore values of approximately −4.6 and were therefore essentially indistinguishable at the docking level. After optimization, their ΔG values were −25.58 and −16.14 kcal mol^−1^, respectively. The optimized Up structure was thus approximately 9.44 kcal mol^−1^ lower in calculated G than the corresponding Down structure under the applied computational model.

The FEXBOA_G–AJUVAC_CD pair therefore demonstrates that quantum-chemical relaxation can reveal an energetic separation that is not evident from the empirical docking score. Similar behaviour was observed for other guests. For example, HAKPUG_G and KOFJEU_G showed a marginal GlideScore preference for MIN_Down, whereas the optimized MIN_Up structure had the lower calculated G.

The complete dataset, however, does not support interpreting DFT refinement as a universal procedure for recovering the crystallographic orientation. Based solely on the formal MIN_Up–MIN_Down energy ordering, MIN_Up had the lower calculated G for 14 of the 20 guests, whereas MIN_Down was lower for six. This corresponds to a 70% orientation agreement based on the formal DFT energy ordering, compared with 65% for the GlideScore-based Top-1 criterion. This modest numerical difference does not support interpreting DFT refinement as a generally more successful predictor of the crystallographic orientation. Rather, the DFT calculations provide a complementary assessment of how selected docking-derived structures behave after unconstrained quantum-chemical relaxation.

The six cases favoring MIN_Down were KUTJUE_G, NILQUV_G, TUXKUS_G, UPULEX_G, VOQDUA_G, and XADHIT_G. The magnitude of this preference varied greatly. Particularly pronounced differences were observed for UPULEX_G, for which MIN_Down was lower by 27.54 kcal mol^−1^, and for KUTJUE_G and NILQUV_G, with differences of 7.50 and 5.37 kcal mol^−1^, respectively. XADHIT_G showed a smaller difference of 2.15 kcal mol^−1^. In contrast, the formal Down preference for TUXKUS_G and VOQDUA_G amounted to only 0.82 and 0.22 kcal mol^−1^, respectively, and these cases should therefore be regarded as nearly degenerate rather than as evidence for a robust energetic preference.

UPULEX_G illustrates particularly well the distinction between different levels of the computational workflow. It showed the highest frequency of crystal-consistent docking poses in the dataset, with 83% classified as Up, yet its optimized MIN_Down structure was considerably lower in calculated G than MIN_Up. XADHIT_G showed a related discrepancy: 80% of its docking poses were Up, while the lowest calculated G among its MIN structures belonged to the Down orientation. The particularly large MIN_Up–MIN_Down difference for UPULEX (27.54 kcal/mol) requires additional caution. The two MIN structures do not represent reversal of the guest within an identical receptor geometry: MIN_Up originated from OFAXID_CD, whereas MIN_Down originated from CIQFIU_CD. Their subsequent unconstrained DFT optimizations therefore involve different initial host geometries and independent relaxation pathways. The resulting energy difference contains contributions from guest orientation together with β-CD deformation, cavity reorganization, intramolecular hydrogen-bond rearrangement, and host–guest complementarity.

The strong dependence on receptor geometry is further illustrated by the UPULEX redocking results. For poses originating from the same matched crystal-derived receptor, the calculated ordering is reversed: REDOCK_Up is 18.10 kcal/mol lower in G than REDOCK_Down, whereas MIN_Down is 27.54 kcal/mol lower than MIN_Up. Therefore, the 27.54 kcal/mol MIN difference should not be interpreted as an intrinsic energetic preference of UPULEX for the Down orientation. Instead, it illustrates the sensitivity of independently optimized β-CD–guest structures to host selection and structural relaxation within the applied gas-phase DFT model.

These observations demonstrate that at least three distinct computational questions must be considered separately: whether a particular orientation is generated, whether it is ranked favorably by GlideScore, and how the selected pose behaves after full quantum-chemical geometry relaxation and thermochemical evaluation. Agreement between these stages occurs for some complexes but is not universal.

Such behaviour is chemically plausible for β-CD inclusion complexes. Reversal of an asymmetric guest does not necessarily destroy the principal stabilizing contacts. The aromatic fragment can remain accommodated within the relatively hydrophobic cavity in both orientations, while the two substituents can establish alternative contacts with groups surrounding the primary and secondary rims. An empirical scoring function can consequently assign comparable values to opposite orientations, whereas relaxation of the β-CD geometry and its hydrogen-bonding network can produce substantially different optimized structures.

### 2.5. The Optimized Crystal-Derived Structure Does Not Necessarily Correspond to the Lowest G Structure Within the Gas-Phase Model

For 19 of the 20 investigated guests, at least one optimized docking-derived structure had a calculated G lower than that of the corresponding optimized crystal-derived reference (ΔG < 0) (Figure 4). The only exception was OFAXID_G, for which the lowest value differed from the crystal-derived reference by only approximately +0.04 kcal mol^−1^ and was therefore effectively indistinguishable at the precision relevant to the present calculations.

This observation should not be interpreted as evidence that the experimentally determined crystal structures are unstable or incorrect. The structures denoted CCDC in the computational analysis no longer represent the complete crystalline systems. Crystallographic water molecules, disorder, and other components of the solid-state environment were removed, leaving only the 1:1 β-CD–guest pair, which was subsequently optimized without geometrical restraints.

The optimized crystal-derived structure therefore represents the result of relaxing an experimental starting geometry within the same simplified gas-phase quantum-chemical model applied to the docking-derived structures. It does not retain the complete energetic environment responsible for stabilization of the original experimental crystal structure.

In particular, crystal packing, explicit water-mediated interactions, and intermolecular hydrogen bonding involving neighboring molecules are absent. Removal of these contributions can change both the preferred β-CD geometry and the relative stability of alternative guest arrangements. A negative ΔG consequently indicates that a docking-derived optimized structure is calculated to be lower in G than the optimized crystal-derived reference within the applied model; it does not demonstrate that the experimental structure is unfavorable under crystallographic conditions.

A further distinction is required when comparing MIN_Up and MIN_Down. Because these structures can originate from different β-CD conformations, their energy difference does not isolate the energetic effect of reversing the guest. It also contains contributions associated with the initial host conformation and with the different structural relaxation pathways of the host, including changes in intramolecular hydrogen bonding, cavity geometry, host deformation, and host–guest complementarity.

REDOCK_Up versus REDOCK_Down provides a conceptually more direct comparison because both docking poses originate from the same experimentally derived host conformation. Nevertheless, the β-CD structures are subsequently allowed to relax independently during DFT optimization. Their final energy difference therefore still represents a comparison between two separately optimized structures rather than the energetic effect of guest reversal within a strictly fixed host.

The thermochemical interpretation also deserves caution because β-CD complexes contain numerous low-frequency modes associated with collective and intermolecular motions. Within a conventional harmonic frequency treatment, these modes can contribute appreciably to calculated thermal and entropic terms. Accordingly, small differences in ΔG, particularly differences substantially below approximately 1 kcal mol^−1^, should not be overinterpreted as robust energetic discrimination. The sign of a small MIN_Up–MIN_Down energy difference should therefore not be interpreted as a definitive assignment of the preferred guest orientation. The observed 14/20 Up-versus-Down count should be regarded as a descriptive result of the applied computational model rather than as a set of quantitatively benchmarked energetic predictions. These uncertainties are compounded by the use of a moderate basis set and by the absence of an implicit or explicit solvent description. Therefore, small Up–Down energy differences should be regarded as model-dependent rather than as quantitatively robust thermodynamic preferences. Future studies employing larger basis sets together with continuum or explicit solvation will be valuable for assessing the robustness of these energetic rankings.

For these reasons, the values reported in Figure 4 are most appropriately treated as relative thermochemical Gibbs free-energy estimates for optimized complexes, rather than as binding free energies or direct experimental free energies of complex formation.

### 2.6. DFT Optimization Can Either Increase or Decrease Structural Similarity to the Crystal-Derived Reference

The binary Up/Down classification establishes whether the direction of inclusion agrees with the crystallographic reference but intentionally ignores more subtle structural differences. Two Up poses may share the same direction while differing considerably in insertion depth, aromatic-ring inclination, lateral displacement, or substituent geometry. Heavy-atom RMSD after host-based alignment was therefore used as a complementary measure of detailed structural similarity.

Guest heavy-atom RMSD was calculated relative to the optimized crystal-derived reference after β-CD host alignment. Figure 5 compares RMSD values before and after DFT geometry optimization for MIN_Up and, where available, REDOCK_Up structures.

For MIN_Up, DFT optimization decreased the RMSD for 10 of the 20 guests, increased it for nine, and produced essentially no change for NILQUV_G at the reported precision. The effect of geometry optimization was therefore almost evenly divided between improved and reduced agreement with the crystal-derived reference.

Several pronounced improvements were nevertheless observed. For KUTJUE_G, the heavy-atom RMSD of MIN_Up decreased from 2.43 to 0.79 Å, corresponding to an improvement of approximately 1.63 Å. The superposition shown in Figure 6 illustrates how geometry optimization moved the docked guest markedly closer to the optimized crystal-derived reference.

A similarly large decrease was observed for QACXEX_G, for which MIN_Up RMSD changed from 5.23 to 3.29 Å, a reduction of 1.94 Å. TUXKUS_G improved from 3.67 to 1.88 Å, while HEGXUM_G decreased from 2.66 to 1.23 Å. FEXBOA_G also showed improved agreement, with RMSD decreasing from 2.80 to 2.02 Å.

For the 16 guests for which a REDOCK_Up pose was available, geometry optimization decreased RMSD in 11 cases and increased it in five. Particularly large improvements were observed for AVUDUS_G, where RMSD decreased from 3.75 to 1.62 Å, and for HEGXUM_G and XADHIT_G, for which the decreases amounted to approximately 1.64 and 1.29 Å, respectively.

The opposite behaviour was also observed. HAKPUG_G showed the largest increase in MIN_Up RMSD, from 2.08 to 4.38 Å. UPULEX_G increased from 1.11 to 3.18 Å, FODBIK_G from 3.39 to 4.27 Å, and VOQDUA_G from 2.60 to 3.59 Å. Among REDOCK_Up structures, FODBEG_G showed a particularly pronounced increase, from 1.33 to 3.98 Å.

These data demonstrate that DFT geometry optimization does not act as a systematic correction toward the crystallographic geometry. The optimization follows the local potential-energy surface defined by the quantum-chemical model and is not restrained toward the experimental coordinates. Consequently, a docking-derived pose may relax toward a geometry more similar to the optimized crystal-derived reference, but it may equally converge toward a structurally different stationary arrangement.

This result also highlights the distinction between energetic and geometric assessment. Lower calculated G does not necessarily imply smaller RMSD, and smaller RMSD does not demonstrate that the corresponding structure has the lowest calculated G. The two descriptors probe fundamentally different properties: RMSD quantifies structural similarity to the selected reference, whereas ΔG compares the thermochemical estimates obtained for independently optimized structures.

Accordingly, RMSD and relative Gibbs free energy should be regarded as complementary rather than interchangeable assessment criteria.

### 2.7. Implications for the Validation of β-Cyclodextrin Docking

Taken together, the present results demonstrate that validation of molecular docking to β-CD cannot be reduced to a single numerical measure. For asymmetric guests, at least four related but distinct properties can be evaluated: orientation sampling, pose ranking, energetic behaviour after refinement, and detailed structural similarity.

The crystal-derived receptor set performed particularly well with respect to orientational sampling. Although individual β-CD conformations frequently failed to produce the crystallographic orientation for a particular guest, the complete set of 20 experimentally derived host conformations generated at least one Up pose for every compound. Moreover, redocking to the matched crystallographic host failed to generate any retained Up pose for four guests. Within this benchmark, host conformational diversity therefore provided a clear benefit at the level of orientational sampling.

Successful sampling did not, however, guarantee successful selection. Only approximately 26% of all retained docking poses were Up, and the globally most favorable GlideScore belonged to the Up orientation for 13 of 20 guests. For several systems, the difference between the best Up and Down scores was negligible. Thus, DFT refinement can provide additional energetic differentiation for selected docking poses, but the resulting ordering remains dependent on the applied quantum-chemical model and should not be regarded as a definitive resolution of near-degenerate cases without further methodological benchmarking.

The comparison between redocking and cross-docking further demonstrates that the β-CD structure extracted from the corresponding experimental complex cannot be assumed to constitute a universally optimal computational receptor. Only three of the 40 orientation-specific global GlideScore minima were obtained using the matched crystal-derived β-CD conformation. Different guests therefore preferred different members of the host conformational set according to the empirical scoring function.

This result argues against relying on a single arbitrarily selected β-CD geometry when directional prediction is the objective. Importantly, the crystal-derived host set used here should not be interpreted as a thermodynamic equilibrium conformational ensemble of β-CD. Rather, it represents a structurally diverse collection of receptor geometries obtained from different crystalline environments. The individual structures may reflect guest-specific effects, hydration, crystal packing, and intermolecular interactions. Accordingly, the purpose of this receptor set is to assess the sensitivity of docking results to host geometry, rather than to reproduce the equilibrium conformational distribution of β-CD.

DFT refinement added a further layer of information but did not remove all ambiguity. Formal comparison of MIN_Up and MIN_Down Gibbs free-energy estimates favoured the Up orientation for 14 of 20 guests, only one more case than the GlideScore criterion. Accordingly, the present data provides no evidence that DFT refinement offers a general improvement in orientation prediction relative to GlideScore ranking. Moreover, some of the DFT differences were small, whereas other systems displayed substantial preference for the directionally inverted structure. Thus, higher-level geometry optimization can resolve selected near-degenerate docking cases, but it should not be regarded as a universal method for identifying the crystallographic orientation.

A similarly heterogeneous response was observed for structural refinement. DFT optimization substantially reduced guest RMSD in several cases, including KUTJUE_G, QACXEX_G, TUXKUS_G, AVUDUS_G, and HEGXUM_G, but increased deviation from the crystal-derived reference in others. The outcome of refinement is therefore strongly system-dependent.

The apparent disagreement between crystallographic orientation, GlideScore, calculated gas-phase G, and RMSD should not in itself be viewed as a failure of the computational workflow. These quantities describe different properties under different physical approximations. The crystal structure represents a particular solid-state arrangement influenced by host–guest interactions together with solvent and lattice effects. GlideScore is an empirical pose-ranking quantity. The DFT-derived G values describe thermochemical estimates for optimized gas-phase structures within the selected quantum-chemical and harmonic models. RMSD, in turn, measures geometric similarity to an optimized structure derived from the crystallographic reference. There is therefore no fundamental requirement for all these criteria to produce identical rankings.

This distinction is especially relevant for β-CD because of its conformational flexibility and directional asymmetry. Two opposite orientations can preserve inclusion of the aromatic core while replacing one set of interactions at the primary and secondary rims with another. Such structures may be difficult to distinguish using an empirical scoring function, while unrestricted host relaxation can further alter their energetic and geometrical relationships.

The present benchmark therefore supports a hierarchical strategy for evaluating β-CD docking. Receptor conformational diversity can first be used to broaden orientational sampling. The presence of a crystal-consistent pose should then be evaluated independently from its numerical GlideScore ranking. Where necessary, selected alternative structures can be subjected to higher-level structural and energetic analysis, while recognizing that such calculations describe the simplified computational model rather than the complete crystallographic environment. Finally, detailed structural metrics such as guest RMSD should complement rather than replace orientation-based analysis.

For asymmetric β-CD guests, the Up/Down criterion is particularly useful in this respect. RMSD addresses how closely a calculated pose reproduces the detailed geometry of the reference complex, whereas Up/Down classification asks whether the guest is inserted into the directionally asymmetric β-CD cavity in the same direction as observed crystallographically. A pose may therefore be crystal-consistent in direction despite a relatively high RMSD, while another may occupy the cavity favorably but be directionally inverted.

Within the present benchmark, the principal challenge was therefore not the absolute inability of the docking procedure to generate crystallographically observed inclusion modes. With sufficient receptor conformational diversity, an Up pose was obtained for every investigated guest. The more difficult problem was selection: reliably distinguishing the crystallographic orientation from a chemically plausible inverted alternative.

Accordingly, the validation of β-CD docking should explicitly separate pose generation from pose ranking and should recognize energetic refinement and geometric similarity as additional, complementary levels of analysis. The present results demonstrate that these criteria are related but not equivalent, and that receptor conformation can play a decisive role in determining both the orientations sampled and their subsequent ranking.

## 3. Materials and Methods

### 3.1. Structure Selection and Preparation

20 complexes containing guests with para-substituted benzene rings and a 1:1 host-to-guest stoichiometry were selected from the Cambridge Structural Database [28]. For each guest, the two substituents were different, enabling the two sides of the guest molecule to be distinguished and its orientation to be assessed unambiguously. The list of selected guest molecules is provided in Table S1 of the Supplementary Materials.

Since guest molecules and β-CD conformations have been extracted from crystal structures deposited in the CSD, they were prepared before being used in docking simulations. For disordered structures, atomic positions with the highest occupancy values were retained. Missing hydrogen atoms were added automatically using Materials Studio Visualizer 2024 [29].

Further, guest structures were prepared using LigPrep with geometry optimization employing the OPLS4 force field, while the original ionization states and specified stereochemistry were retained. Because crystal structures often lack hydrogen atoms, β-CD conformations have been prepared using the Protein Preparation Workflow. Solely the hydrogen atoms were minimized with the OPLS4 force field, whereas the coordinates of all other atoms were retained from the crystal structures. Subsequently, each prepared guest molecule was docked to every prepared β-CD conformation, thereby performing so-called cross-docking and redocking.

### 3.2. Molecular Docking

Molecular docking was performed using Glide implemented in the Maestro Schrödinger package (version 13.7.125) [30]. A separate receptor grid was generated for each β-CD conformation. The docking grid was defined to encompass the entire β-CD molecule, thereby allowing ligand sampling throughout the complete host cavity as well as in the vicinity of both the primary and secondary rims. No additional spatial constraints were imposed to favor either guest orientation. Docking calculations were carried out in standard precision (SP) mode using flexible ligand sampling and post-docking minimization with the OPLS4 force field [31]. Unless otherwise stated, the default Glide settings were used for receptor grid generation and docking, including the default van der Waals scaling parameters. Guest structures were prepared with LigPrep without generation of alternative ionization states; the ionization state, formal charge, and specified stereochemistry of each crystal-derived guest structure were retained. The maximum number of generated poses per ligand was set to 200, and up to 20 poses with the most favorable GlideScore values were retained from each docking calculation. In total, 7916 complexes obtained from docking 20 guests into 20 β-CD conformations were subjected to subsequent orientation analysis.

### 3.3. Name Convention

Structure names were generated according to the following convention:

The first part of the name corresponds to the refcode of the crystal structure in the CSD from which the guest or β-CD conformation has been extracted. The suffixes “_G” and “_CD” indicate guest and β-CD structure, respectively. For example, a complex composed of a guest extracted from the crystal structure with the refcode FEXBOA and a β-CD conformation extracted from the structure with the refcode AJUVAC is denoted as FEXBOA_G-AJUVAC_CD.

“REDOCK” refers to a complex from docking in which the guest and β-CD are derived from the same crystal structure and have the most favorable docking score. Whereas “MIN” corresponds to a complex with the most favorable docking score among all simulations performed using the tested β-CD conformations, for each guest. Suffixes “Up” and “Down” indicate if the guest orientation is as in the crystal complex or the opposite, respectively.

Optimized complexes derived from crystal structures were assigned the label “CCDC”.

### 3.4. DFT Calculations

For each guest, selected complexes were subjected to further optimization at the DFT level. These included the experimental complex obtained from the crystal structure, the complexes with the most favorable docking scores for the Up and Down guest orientations, denoted MIN_Up and MIN_Down, respectively, and the redocked complexes with the most favorable docking scores for each orientation, REDOCK_Up and REDOCK_Down. Additionally, one representative Up and Down pair with nearly degenerate GlideScore was selected for illustrative DFT analysis, namely the FEXBOA_G-AJUVAC_CD complexes. DFT calculations were performed using Gaussian 16 Revision C.01 software with the B3LYP functional and 6-31G(d,p) basis set with Grimme D3(BJ) dispersion corrections [32]. Twenty β-cyclodextrin conformations extracted from selected CSD complexes used for cross-docking and redocking were DFT-optimized to compare their relative energies.

Frequency calculations were performed to confirm the absence of imaginary frequencies, thereby verifying that the optimized structures are in a stationary state and enabling thermodynamic analysis.

### 3.5. Host-Based Alignment and Guest Heavy-Atom RMSD Calculations

The DFT-optimized experimental complex was used as the reference structure. Alignment was performed using CDGA Align 1.0, a Cyclodextrin Guest Analysis tool developed in our laboratory [33]. The algorithm, implementation, and validation of CDGA Align have been described in a dedicated methodological study that has been accepted for publication [33]. Briefly, structural superposition was performed using the β-CD host atoms so that the guest coordinates did not influence the alignment. Following host alignment, the heavy-atom RMSD of the guest was calculated relative to the DFT-optimized crystal-derived reference structure. The aligned complexes were additionally inspected in Materials Studio 2024 to verify host superposition and guest-atom correspondence. An example of the resulting superposition is shown in Figure 6.

### 3.6. Guest Orientation Assessment

For all 7916 docked poses and all DFT-optimized complexes, guest orientation was assessed using CDGA Align 1.0 according to the procedure described and validated in the accepted methodological study [33]. An orientation consistent with that observed in the corresponding reference crystal structure was assigned a value of 1 and denoted Up, whereas the directionally inverted orientation was assigned a value of 0 and denoted Down. All automated assignments were independently inspected by two researchers to verify the correctness of the classification.

### 3.7. Statistical Analysis

Because multiple docking poses were generated for each guest–receptor combination, individual retained poses were not treated as statistically independent observations. The overall Up/Down frequencies calculated from the complete set of 7916 poses were therefore used only as descriptive pose-level statistics. For guest-level analysis, the proportion of Up poses was calculated separately for each of the 20 guests across the complete set of β-CD receptor conformations. The mean, median, and interquartile range of these guest-specific proportions were determined, and a non-parametric 95% bootstrap confidence interval for the mean was calculated by resampling the 20 guests. For the Top-1 orientation-recovery analysis, each guest constituted one observation, and the 95% confidence interval for the proportion of guests for which the globally best-scoring pose had the crystallographically consistent orientation was calculated using the Wilson method. Orientation sampling and ranking-based orientation recovery were evaluated separately. Sampling success was defined as the presence of at least one crystal-consistent Up pose for a given guest among all poses retained across the complete set of crystal-derived β-CD receptors. For the Top-N analysis, all retained poses obtained for each guest across the 20 receptor conformations were pooled and ranked in ascending order of GlideScore. Top-N recovery was considered successful when at least one Up pose occurred among the N highest-ranked poses. Top-1, Top-2, Top-3, and Top-5 recovery rates were calculated at the guest level. For each Top N orientation recovery rate, the 95% confidence interval was calculated using the Wilson score method and provided in Table S3. For descriptive comparison, a simple 50:50 random-orientation baseline was considered, for which the probability of obtaining at least one Up orientation among N poses is $1-{\left(0.5\right)}^{N}$.

## 4. Conclusions

The present study provides a systematic validation of molecular docking for predicting the directional orientation of asymmetric guests within the β-cyclodextrin cavity. By using 20 β-CD host structures derived from crystallographic complexes deposited in the CSD and 20 crystallographically characterized 1,4-disubstituted benzene guests, both redocking and cross-docking could be evaluated within a common and structurally controlled benchmark.

The results demonstrate that host (β-CD) conformation has a pronounced influence on the orientation of the docked guest. Individual β-CD structures differed substantially in their ability to generate poses consistent with the crystallographic inclusion mode, and no single host conformation performed optimally for all guests. Importantly, however, the complete set of crystal-derived β-CD receptors generated at least one crystal-consistent Up pose for every guest investigated. Thus, the principal advantage of using multiple structurally diverse receptor geometries in this system was improved orientational sampling, rather than guaranteed identification of the experimentally observed pose.

Pose generation and pose ranking were shown to represent distinct aspects of docking performance. Although the complete receptor set sampled at least one crystal-consistent Up pose for every investigated guest, the Top-1 orientation-recovery rate based on GlideScore ranking was only 65.0% (13/20). Recovery increased to 95.0% for Top-2 and 100% for Top-3, indicating that the experimentally observed orientation was generally present among the highest-ranked solutions but was not always selected as the single best-scoring pose. Thus, successful orientational sampling should not be interpreted as equivalent to successful top-ranked prediction.

DFT refinement provided an additional, model-dependent level of structural and energetic analysis but did not improve orientation prediction in a systematic manner. For 14 of the 20 guests, the optimized MIN_Up structure had a lower calculated Gibbs free energy than the corresponding MIN_Down structure. This 14/20 result represents a formal sign-based comparison within the applied DFT model and should not be interpreted as a set of quantitatively validated energetic preferences, particularly for near-degenerate Up/Down pairs. Likewise, geometry optimization improved agreement with the optimized crystal-derived reference for some complexes but increased guest RMSD for others. Energetic ranking and structural similarity should therefore be regarded as complementary rather than interchangeable validation criteria.

The results further emphasize that gas-phase DFT-optimized structures and crystallographic complexes describe different physical situations. Crystal packing, explicit water molecules, intermolecular hydrogen bonding, and other solid-state contributions are absent from the simplified computational model. Accordingly, a docking-derived optimized structure with a lower calculated Gibbs free energy than the optimized crystal-derived reference should not be interpreted as contradicting the experimental structure. Future work should examine whether the relative energetic ordering of alternative guest orientations is preserved when larger basis sets and explicit or continuum solvation models are employed. Accordingly, the present calculations do not establish whether the gas-phase Up/Down energetic ordering would be preserved upon solvation.

Overall, the benchmark shows that the major challenge in β-CD docking is not necessarily the inability to generate the crystallographically observed inclusion direction, but rather the reliable selection of that orientation from competing alternatives. For asymmetric cyclodextrin guests, validation should therefore distinguish explicitly between orientational sampling, scoring, higher-level energetic refinement, and detailed geometrical agreement. The Up/Down classification introduced here provides a simple and chemically meaningful complement to conventional RMSD analysis and highlights the importance of receptor conformational diversity in computational studies of β-CD host–guest complexes.

## Figures and Tables

**Figure 1:**
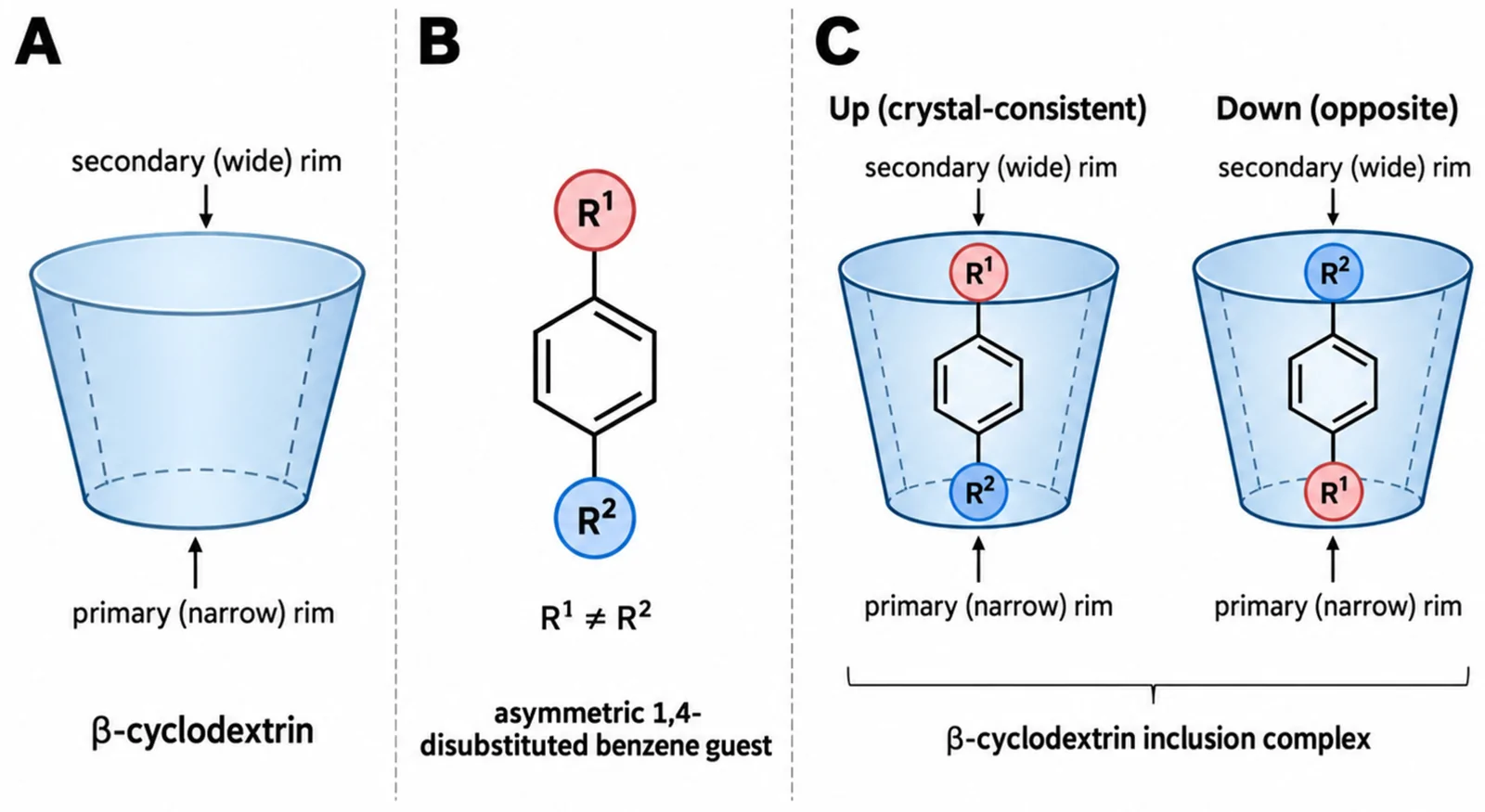
Simplified two-dimensional schematic representation of the host–guest systems investigated in this study: (**A**) β-cyclodextrin with the wider secondary rim and the narrower primary rim indicated; (**B**) the general asymmetric 1,4-disubstituted benzene guest motif used in the benchmark (R^1^ ≠ R^2^); and (**C**) schematic illustration of the two possible guest orientations within the β-cyclodextrin cavity, denoted Up (crystal-consistent orientation) and Down (opposite orientation).

**Figure 2:**
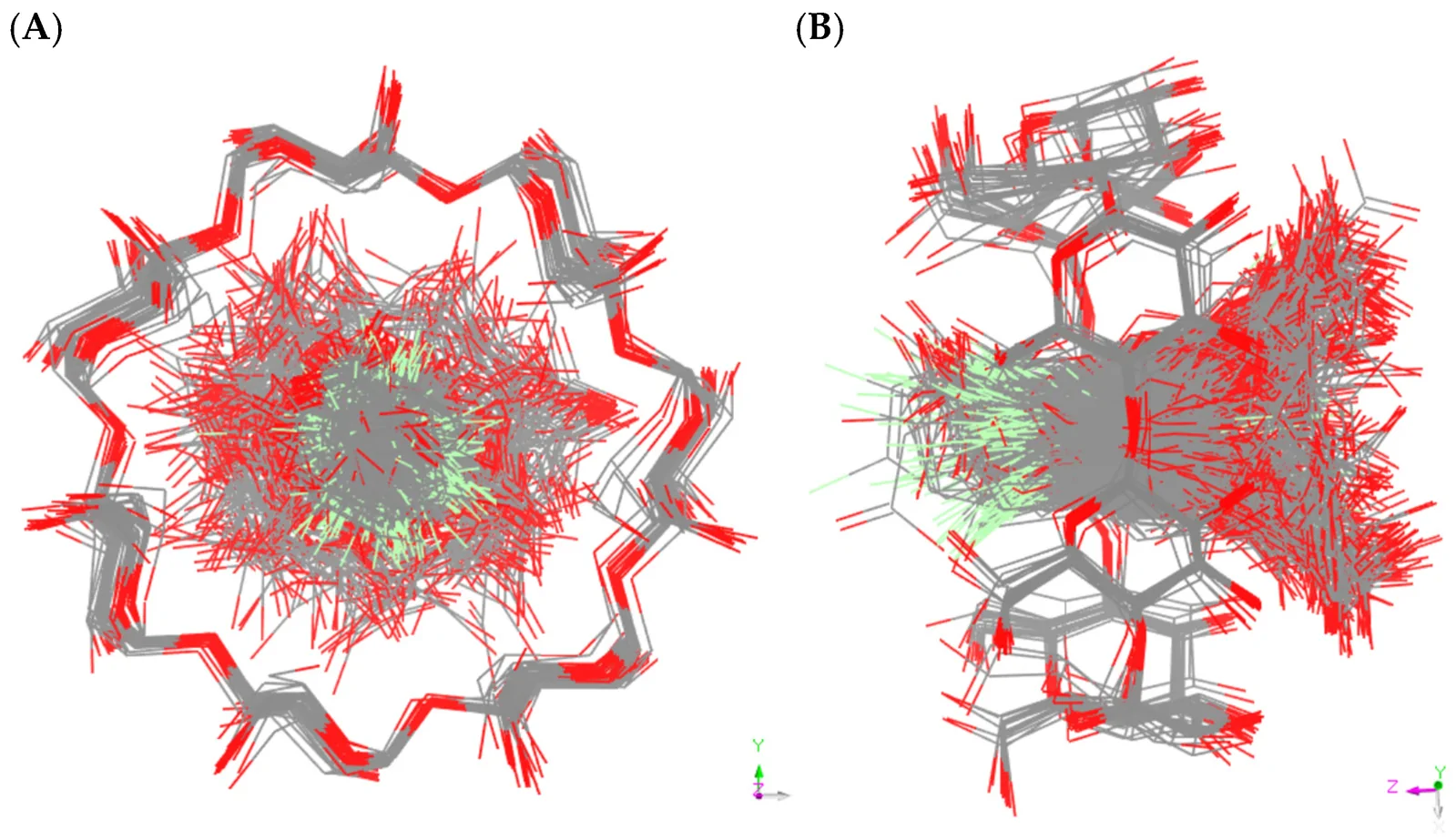
Superposition of docking poses for FEXBOA_G viewed along the β-CD cavity axis (**A**) and perpendicular to the cavity axis (**B**). Hydrogen atoms are hidden for clarity.

**Figure 3:**
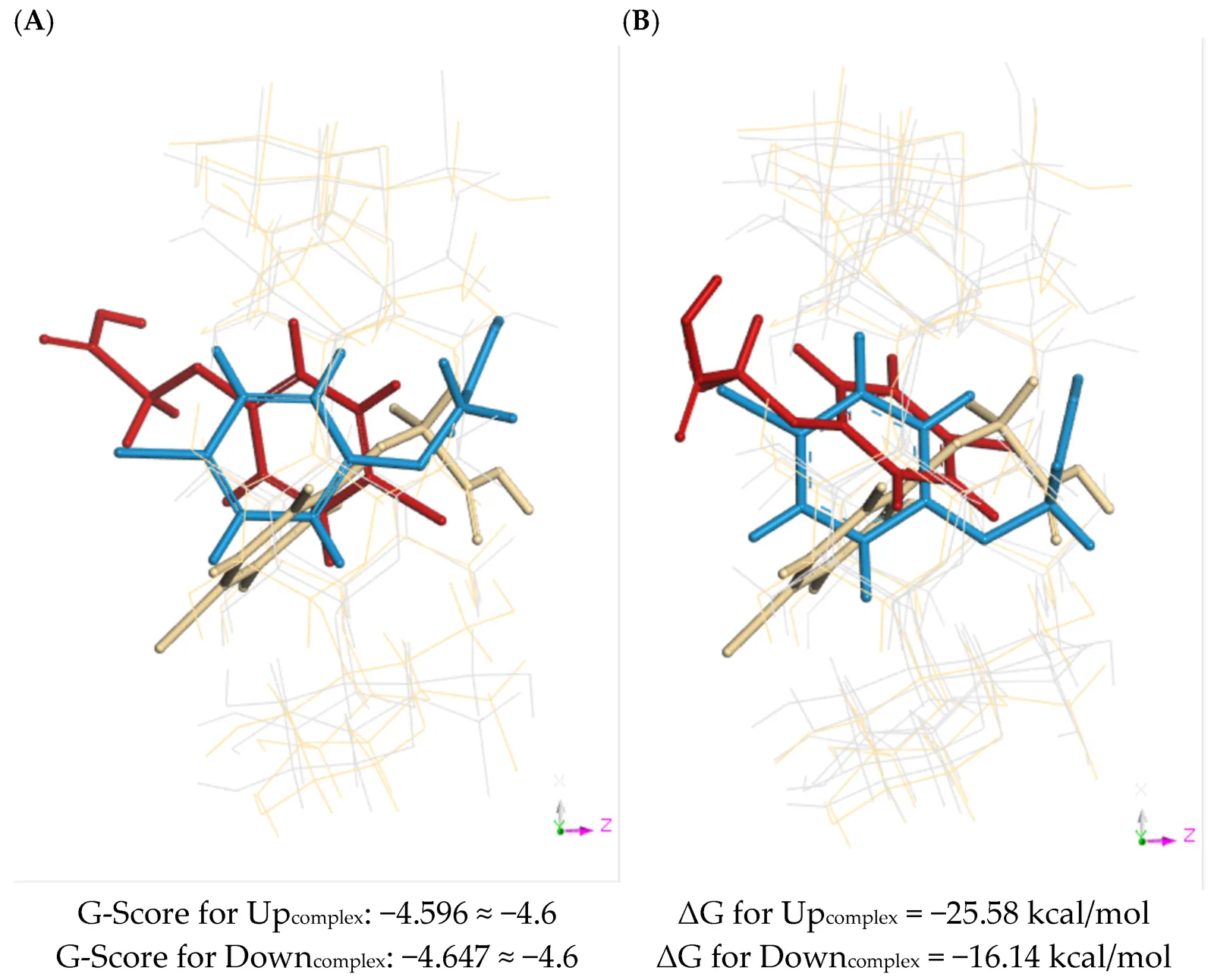
Complexes of FEXBOA_G-AJUVAC_CD before (**A**) and after DFT geometry optimization (**B**), obtained by molecular docking in comparison to the optimized crystal structure of the FEXBOA complex (yellow). Pose Up is shown in blue and pose Down in red. Values of ΔG were provided with reference to the optimized crystal structure of the FEXBOA complex.

**Figure 4:**
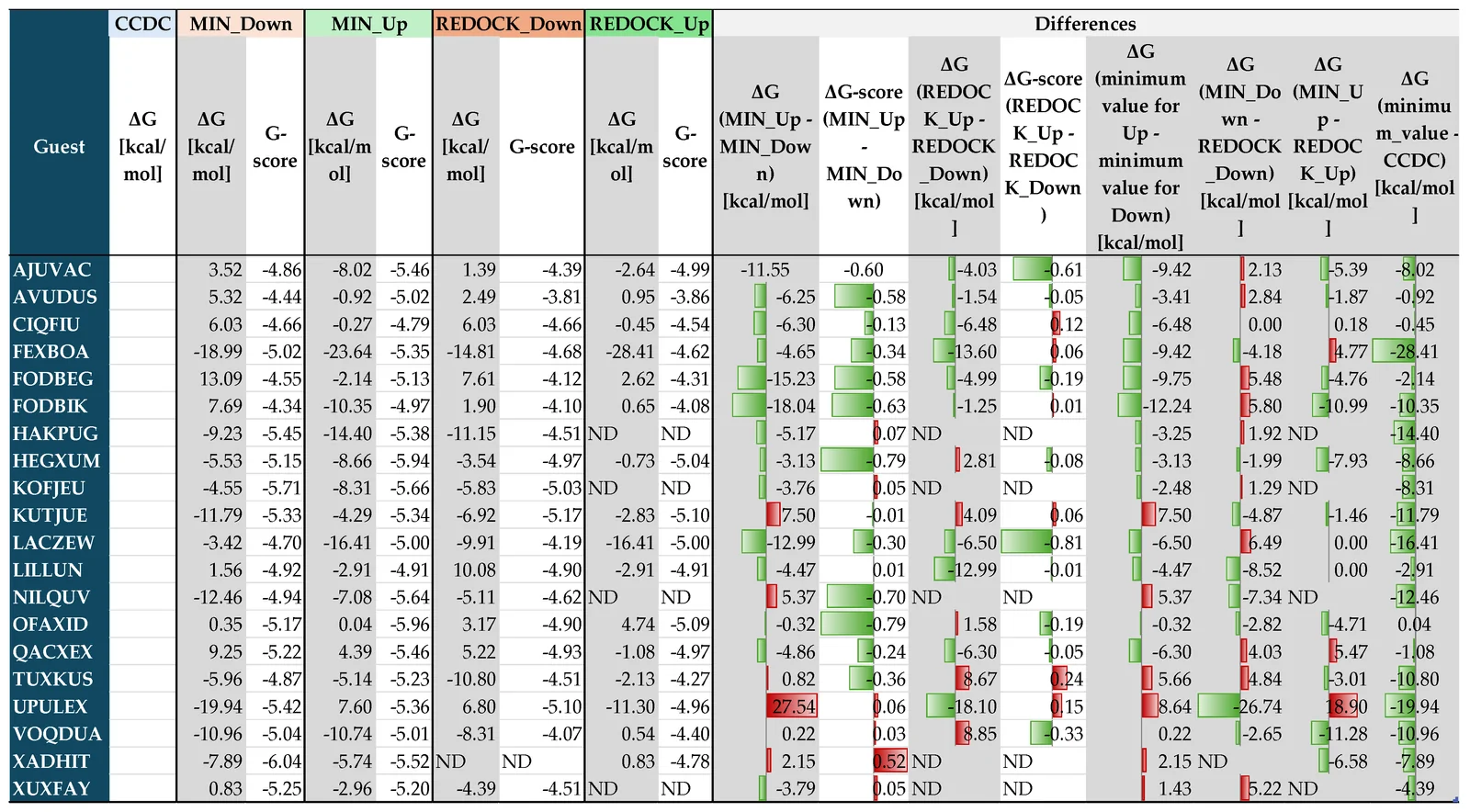
Summary of molecular docking and DFT calculations, including G-Score and Gibbs free energy values, for the complexes selected for geometry optimization at the DFT level. The Gibbs free energy values are reported relative to the optimized crystal structure of the complex corresponding to each refcode. For some systems, MIN and REDOCK correspond to the same docking pose but were optimized independently by DFT, resulting in negligible numerical differences. For consistency, a single value is reported in the table.

**Figure 5:**
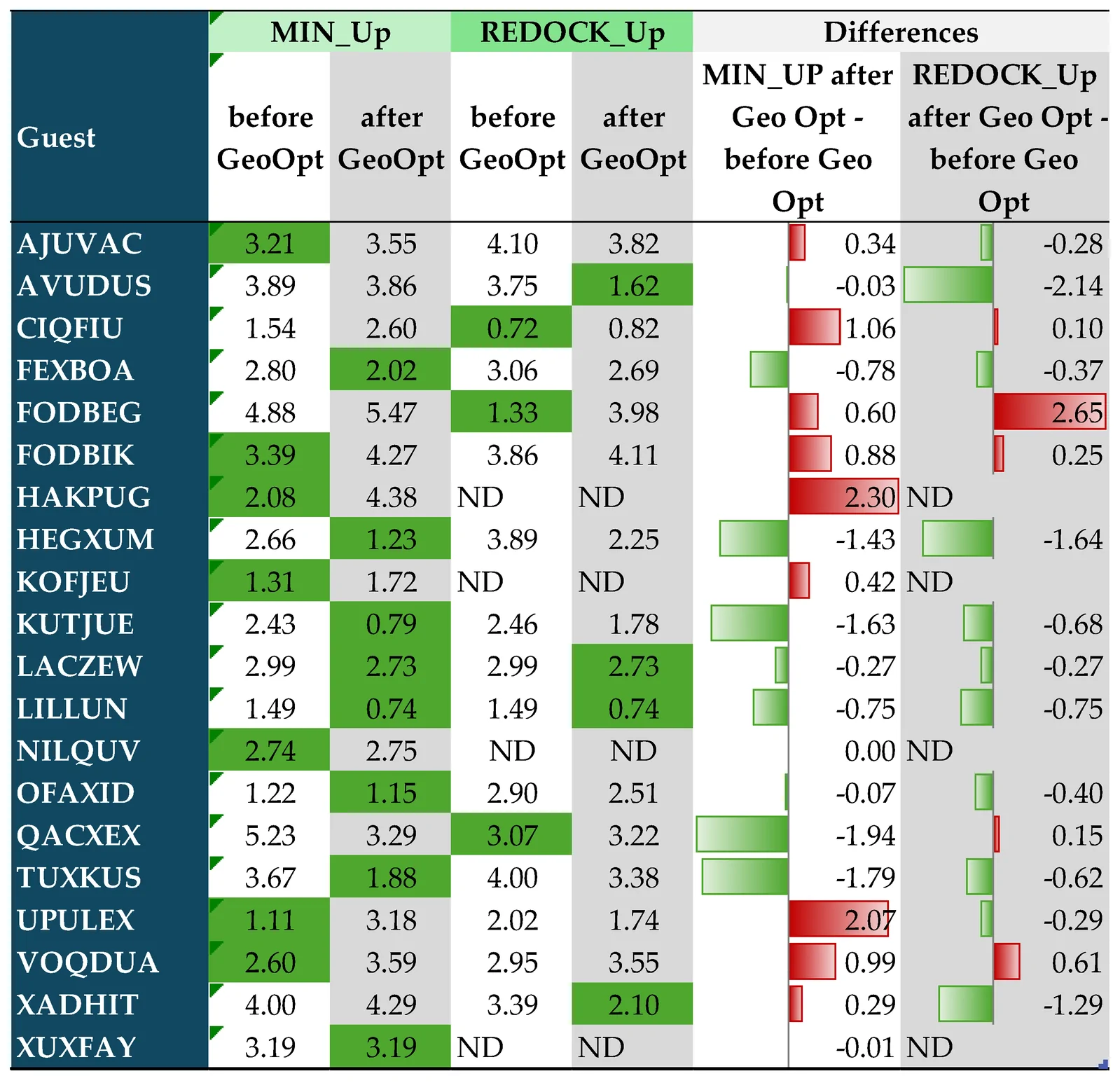
Heavy-atom RMSD values for each guest before and after geometry optimization (GeoOpt) were calculated relative to the optimized crystal structure for the MIN_Up and REDOCK_Up complexes. The lowest RMSD value for each guest was marked in green. The difference between the RMSD values after and before geometry optimization was also calculated.

**Figure 6:**
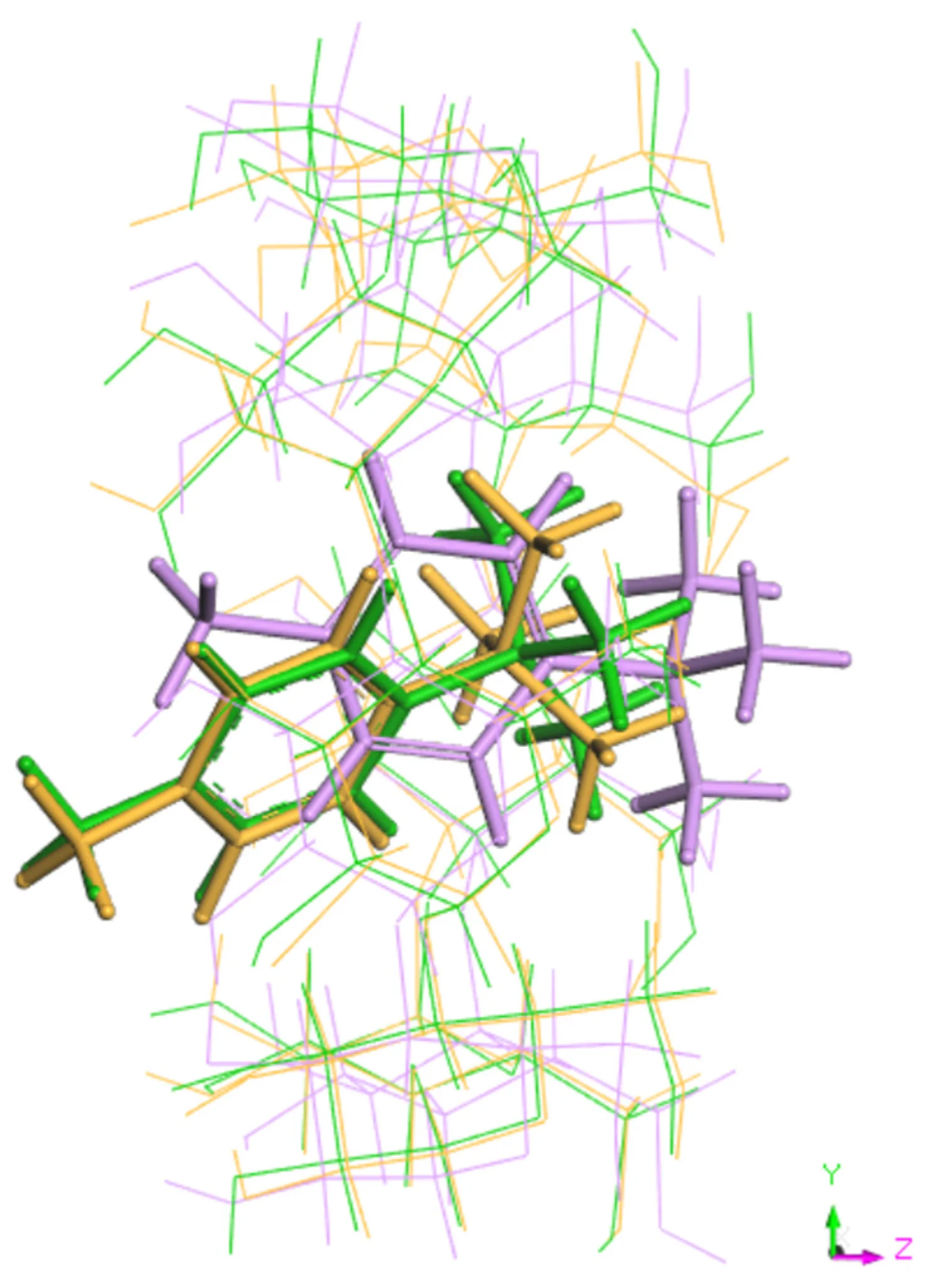
Superposition of the MIN_Up complex for KUTJUE_G before and after DFT geometry optimization, shown in violet and green, respectively. The optimized complex derived from the crystal structure of KUTJUE is shown in yellow.

**Table 1:** Maximum and minimum GlideScore (G-Score) values and their ranges for each guest.

Guest	Max of G-Score	Min of G-Score	Range
AJUVAC_G	−0.22	−5.46	5.24
AVUDUS_G	−3.35	−5.02	1.67
CIQFIU_G	−2.96	−4.79	1.83
FEXBOA_G	−3.59	−5.35	1.76
FODBEG_G	−3.49	−5.13	1.65
FODBIK_G	−3.48	−4.97	1.49
HAKPUG_G	−3.86	−5.45	1.58
HEGXUM_G	−4.21	−5.94	1.74
KOFJEU_G	−4.34	−5.71	1.37
KUTJUE_G	−4.40	−5.34	0.94
LACZEW_G	−3.30	−5.00	1.70
LILLUN_G	−2.89	−4.92	2.03
NILQUV_G	−3.94	−5.64	1.71
OFAXID_G	−4.35	−5.96	1.61
QACXEX_G	−4.38	−5.46	1.08
TUXKUS_G	−3.57	−5.23	1.66
UPULEX_G	−4.12	−5.42	1.30
VOQDUA_G	−3.29	−5.04	1.76
XADHIT_G	−2.78	−6.04	3.27
XUXFAY_G	−3.84	−5.25	1.42

**Table 2:** Matrix showing the percentage of Up complexes for each β-CD conformation and guest. The β-CD conformations are arranged in ascending order of Gibbs free energy, and the guest order was adjusted accordingly.

ΔG β-CD [kcal/mol]		Guest	UPULEX_G	HAKPUG_G	FODBEG_G	OFAXID_G	FODBIK_G	XUXFAY_G	KOFJEU_G	QACXEX_G	AJUVAC_G	AVUDUS_G	CIQFIU_G	HEGXUM_G	VOQDUA_G	TUXKUS_G	NILQUV_G	LILLUN_G	LACZEW_G	KUTJUE_G	XADHIT_G	FEXBOA_G	All Guests
	β-CD																						
	UPULEX_CD		70%	0%	0%	15%	0%	25%	0%	5%	25%	30%	70%	0%	5%	0%	0%	15%	0%	45%	95%	0%	20%
6.32	HAKPUG_CD		100%	0%	0%	5%	0%	5%	5%	10%	15%	0%	5%	0%	5%	0%	0%	10%	0%	50%	90%	0%	15%
7.49	FODBEG_CD		85%	15%	25%	30%	10%	0%	25%	30%	15%	25%	75%	0%	10%	0%	10%	30%	0%	55%	90%	0%	27%
7.68	OFAXID_CD		100%	10%	0%	20%	0%	80%	50%	5%	0%	0%	5%	35%	20%	10%	50%	20%	0%	40%	35%	40%	26%
7.81	FODBIK_CD		95%	5%	10%	25%	5%	25%	50%	20%	13%	5%	50%	25%	15%	20%	5%	20%	5%	40%	80%	0%	26%
8.06	XUXFAY_CD		100%	0%	5%	0%	5%	0%	10%	0%	0%	0%	40%	0%	10%	0%	0%	45%	0%	30%	80%	0%	16%
8.06	KOFJEU_CD		100%	0%	0%	5%	0%	0%	0%	0%	0%	5%	55%	0%	5%	0%	0%	20%	0%	30%	90%	0%	16%
8.95	QACXEX_CD		60%	20%	10%	50%	0%	35%	30%	25%	25%	25%	65%	20%	25%	45%	16%	15%	0%	65%	65%	0%	30%
9.26	AJUVAC_CD		80%	15%	40%	55%	35%	25%	95%	30%	28%	65%	65%	33%	40%	0%	17%	40%	10%	35%	65%	10%	39%
9.54	AVUDUS_CD		95%	0%	60%	50%	40%	0%	60%	30%	44%	45%	40%	6%	10%	5%	20%	15%	0%	35%	80%	0%	32%
10.22	CIQFIU_CD		30%	35%	60%	70%	70%	5%	95%	75%	50%	85%	35%	89%	10%	65%	65%	75%	5%	20%	75%	0%	51%
11.01	HEGXUM_CD		80%	0%	5%	15%	5%	0%	60%	15%	8%	15%	55%	32%	10%	0%	0%	35%	0%	40%	100%	0%	24%
11.01	VOQDUA_CD		90%	0%	5%	10%	0%	0%	70%	25%	0%	5%	35%	35%	20%	0%	0%	55%	0%	40%	90%	0%	24%
11.28	TUXKUS_CD		95%	0%	5%	5%	0%	5%	5%	10%	0%	0%	30%	0%	0%	5%	0%	15%	0%	40%	100%	0%	16%
11.61	NILQUV_CD		100%	0%	0%	10%	0%	0%	10%	0%	10%	5%	15%	0%	5%	0%	0%	30%	0%	30%	100%	0%	16%
12.74	LILLUN_CD		75%	5%	15%	25%	10%	0%	45%	15%	27%	20%	70%	5%	5%	0%	5%	45%	0%	15%	95%	0%	24%
13.42	LACZEW_CD		60%	50%	45%	60%	10%	90%	90%	25%	29%	10%	45%	80%	50%	70%	80%	15%	85%	50%	25%	95%	54%
14.15	KUTJUE_CD		95%	0%	5%	5%	0%	0%	30%	5%	0%	5%	35%	10%	10%	0%	0%	30%	0%	40%	70%	0%	17%
16.39	XADHIT_CD		100%	0%	0%	10%	0%	0%	0%	5%	15%	0%	20%	0%	15%	0%	0%	20%	0%	30%	100%	0%	16%
17.03	FEXBOA_CD		45%	10%	45%	60%	45%	45%	70%	35%	35%	55%	25%	25%	10%	5%	25%	20%	0%	40%	75%	15%	34%
	All β-CDs		83%	8%	17%	26%	12%	17%	40%	18%	18%	20%	42%	19%	14%	11%	15%	29%	5%	39%	80%	8%	26%

To facilitate the analysis of the data in the table, the 3-color scale ranging from 0% to 100% was applied consistently across the entire dataset. Values of 0%, 50%, and 100% are represented by red, yellow, and green, respectively, with intermediate values colored proportionally according to their position within this fixed range.

**Table 3:** Examples of guests with the number of complexes formed for each guest orientation, the percentage of Up complexes, and the schematic representation of Up complexes.

Schematic Representation of Up Complex			Guest	Number of Up Complexes	Number of Down Complexes	Percentage of Up Complexes
Wide rim of β-CD	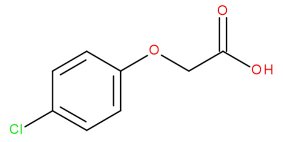	Narrow rim of β-CD	FEXBOA_G	32	368	8%
	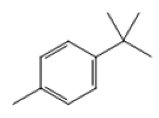		KUTJUE_G	154	246	39%
	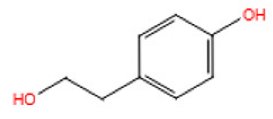		CIQFIU_G	167	233	42%
	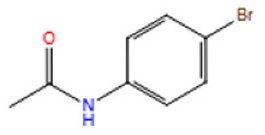		XADHIT_G	320	80	80%

**Table 4:** Minimum docking score values for the Up (1) and Down (0) guest orientations across all guests and β-CD conformations. For each guest and orientation, the most favourable docking score is highlighted in green, whereas the least favourable score is highlighted in red. The β-CD conformations are arranged in ascending order of Gibbs free energy starting from the left, and the guest order has been adjusted accordingly.

	β-CD	Up/Down	UPULEX_CD	HAKPUG_CD	FODBEG_CD	OFAXID_CD	FODBIK_CD	XUXFAY_CD	KOFJEU_CD	QACXEX_CD	AJUVAC_CD	AVUDUS_CD	CIQFIU_CD	HEGXUM_CD	VOQDUA_CD	TUXKUS_CD	NILQUV_CD	LILLUN_CD	LACZEW_CD	KUTJUE_CD	XADHIT_CD	FEXBOA_CD	All CDs
Guest																							
UPULEX_G		0	−5.10	-	−4.64	-	−4.76	-	-	−4.86	−4.63	−4.47	−5.42	−4.51	−4.46	−4.12	-	−5.13	−5.02	−4.43	-	−5.07	−5.42
		1	−4.96	−4.90	−4.78	−5.36	−4.97	−4.99	−4.90	−4.90	−4.90	−4.58	−5.01	−4.96	−4.99	−5.01	−4.97	−5.02	−5.19	−4.97	−4.91	−4.91	−5.36
HAKPUG_G		0	−4.60	−4.51	−4.78	−4.80	−4.60	−4.78	−4.62	−4.57	−4.61	−4.78	−5.45	−4.45	−4.58	−4.68	−4.54	−4.88	−4.33	−4.67	−4.69	−4.55	−5.45
		1	-	-	−5.09	−4.72	−4.53	-	-	−4.69	−4.45	-	−5.25	-	-	-	-	−4.40	−5.38	-	-	−4.44	−5.38
FODBEG_G		0	−4.18	−4.15	−4.12	−4.10	−4.12	−4.06	−4.00	−4.01	−4.07	−3.73	−4.55	−3.92	−3.93	−4.09	−4.18	−4.34	−4.05	−4.14	−4.12	−4.07	−4.55
		1	-	-	−4.31	-	−3.93	−3.89	-	−4.09	−4.90	−4.34	−5.13	−3.93	−4.25	−3.96	-	−4.20	−4.93	−3.77	-	−4.68	−5.13
OFAXID_G		0	−5.17	−4.93	−5.01	−4.90	−5.10	−4.88	−4.94	−4.86	−4.98	−4.55	−5.09	−4.80	−4.71	−4.99	−5.07	−5.15	−4.79	−4.96	−4.97	−4.84	−5.17
		1	−5.07	−4.88	−5.08	−5.09	−5.62	-	−4.83	−5.50	−5.43	−5.14	−5.96	−4.64	−4.67	−5.01	−5.01	−5.55	−5.57	−4.79	−4.90	−5.37	−5.96
FODBIK_G		0	−4.16	−4.13	−4.21	−4.12	−4.10	−3.96	−4.00	−4.01	−4.04	−3.78	−4.11	−3.88	−3.94	−4.06	−4.16	−4.34	−3.99	−4.09	−4.12	−4.00	−4.34
		1	-	-	−4.29	-	−4.08	−3.86	-	-	−4.48	−4.37	−4.97	−3.77	-	-	-	−4.57	−4.48	-	-	−4.42	−4.97
XUXFAY_G		0	−4.63	−4.51	−4.55	−4.38	−4.57	−4.51	−4.45	−4.55	−4.61	−4.88	−4.96	−4.44	−4.41	−4.61	−4.65	−5.25	−4.40	−4.58	−4.51	−4.36	−5.25
		1	−4.65	−4.49	-	−5.11	−4.53	-	-	−4.36	−4.70	-	−4.57	-	-	−4.51	-	-	−5.20	-	-	−4.25	−5.20
KOFJEU_G		0	−4.82	−5.16	−4.81	−5.01	−5.32	−5.14	−5.03	−4.94	−4.78	−5.06	−5.71	−5.06	−5.23	−5.16	−5.08	−5.30	−5.01	−5.21	−5.01	−5.33	−5.71
		1	-	−4.50	−5.02	−4.87	−5.19	−4.55	-	−5.21	−5.48	−5.03	−5.66	−5.11	−5.04	−4.74	−4.56	−5.32	−5.25	−5.20	-	−5.20	−5.66
QACXEX_G		0	−5.16	−5.04	−5.15	−5.05	−5.09	−4.95	−4.98	−4.93	−5.16	−4.80	−5.04	−4.94	−4.94	−5.08	−4.97	−5.22	−5.13	−5.10	−5.03	−4.97	−5.22
		1	−5.11	−4.89	−5.10	−4.75	−5.26	-	-	−4.97	−5.07	−4.75	−5.46	−4.89	−4.87	−5.15	-	−5.08	−5.04	−4.81	−4.91	−4.95	−5.46
AJUVAC_G		0	−4.44	−4.32	−4.40	−4.86	−4.47	−4.40	−4.36	−4.31	−4.39	−4.02	−4.65	−4.30	−4.27	−4.46	−4.45	−4.70	−4.84	−4.48	−4.39	−4.37	−4.86
		1	−5.30	−4.08	−5.00	-	−5.16	-	-	−5.08	−4.99	−4.76	−5.46	−3.48	-	-	−4.23	−5.27	−4.83	-	−4.24	−4.98	−5.46
AVUDUS_G		0	−4.23	−4.00	−4.09	−4.38	−4.13	−3.97	−3.99	−3.98	−4.04	−3.81	−4.44	−3.88	−3.87	−4.10	−4.15	−4.41	−4.39	−4.08	−4.12	−4.07	−4.44
		1	−4.60	-	−4.05	-	−4.00	-	−3.75	−4.35	−4.36	−3.86	−5.02	−3.86	−3.87	-	−3.85	−4.59	−4.28	−3.79	-	−4.48	−5.02
CIQFIU_G		0	−4.09	−3.51	−4.21	−4.04	−4.10	−3.41	−3.41	−4.10	−4.26	−4.08	−4.66	−3.90	−4.07	−3.57	−3.60	−4.30	−4.14	−4.03	−3.40	−4.10	−4.66
		1	−4.23	−3.41	−4.15	−3.88	−4.31	−3.54	−3.63	−4.09	−4.48	−3.88	−4.54	−3.74	−3.72	−3.45	−3.71	−4.79	−4.15	−3.81	−3.83	−4.00	−4.79
HEGXUM_G		0	−4.90	−5.10	−4.89	−4.79	−4.90	−4.96	−4.81	−4.59	−5.00	−4.66	−4.74	−4.97	−4.68	−4.93	−5.15	−4.98	−4.68	−5.03	−5.00	−4.75	−5.15
		1	-	-	-	−5.11	−4.99	-	-	−4.91	−5.21	−4.40	−5.70	−5.04	−5.02	-	-	−4.85	−5.94	−4.66	-	−5.14	−5.94
VOQDUA_G		0	−4.24	−4.09	−4.24	−4.82	−4.32	−4.07	−4.24	−4.04	−4.40	−4.58	−4.72	−4.20	−4.07	−4.19	−4.20	−5.04	−4.29	−4.27	−4.16	−4.08	−5.04
		1	−4.40	−3.79	−3.89	−4.69	−4.31	−3.93	−3.90	−4.54	−4.58	−4.02	−4.55	−4.47	−4.40	-	−3.81	−4.34	−5.01	−4.30	−4.04	−4.20	−5.01
TUXKUS_G		0	−4.40	−4.58	−4.33	−4.33	−4.41	−4.42	−4.34	−4.17	−4.40	−4.72	−4.19	−4.27	−4.38	−4.51	−4.44	−4.87	−4.27	−4.55	−4.51	−4.41	−4.87
		1	-	-	-	−4.15	−4.91	-	-	−4.77	-	−4.17	−4.97	-	-	−4.27	-	-	−5.23	-	-	−4.33	−5.23
NILQUV_G		0	−4.94	−4.64	−4.64	−4.82	−4.74	−4.69	−4.63	−4.59	−4.65	−4.28	−4.92	−4.53	−4.51	−4.73	−4.62	−4.92	−4.83	−4.72	−4.65	−4.70	−4.94
		1	-	-	−4.78	−5.06	−4.96	-	-	−4.73	−4.91	−4.62	−5.56	-	-	-	-	−4.85	−5.64	-	-	−4.99	−5.64
LILLUN_G		0	−4.92	−4.36	−4.28	−4.14	−4.53	−3.94	−3.88	−4.55	−4.37	−3.74	−4.08	−4.20	−4.02	−4.06	−4.02	−4.90	−4.73	−3.97	−4.16	−4.43	−4.92
		1	−3.92	−3.80	−4.36	−3.88	−4.04	−3.63	−3.37	−3.84	−4.42	−4.19	−4.84	−3.68	−4.28	−3.94	−3.93	−4.91	−3.80	−3.92	−3.83	−4.11	−4.91
LACZEW_G		0	−4.12	−4.33	−4.46	−4.21	−4.30	−4.44	−4.35	−4.52	−4.32	−4.22	−4.68	−4.26	−4.27	−4.30	−4.43	−4.70	−4.19	−4.39	−4.26	−4.58	−4.70
		1	-	-	-	-	−3.79	-	-	-	−4.28	-	−4.27	-	-	-	-	-	−5.00	-	-	-	−5.00
KUTJUE_G		0	−5.25	−5.05	−5.20	−5.08	−5.16	−5.03	−5.01	−4.98	−5.33	−4.88	−5.33	−5.03	−4.99	−5.17	−5.03	−5.30	−4.94	−5.17	−5.06	−5.19	−5.33
		1	−5.24	−5.24	−5.17	−5.05	−5.22	−5.09	−5.13	−5.15	−5.19	−4.93	−5.28	−5.11	−5.03	−5.32	−5.34	−5.32	−5.06	−5.10	−5.18	−5.12	−5.34
XADHIT_G		0	−3.26	−4.75	−3.30	−5.31	−5.02	−4.69	−4.67	−5.29	−5.20	−4.62	−5.67	-	−4.84	-	-	−4.97	−6.04	−4.88	-	−5.18	−6.04
		1	−4.87	−4.76	−4.89	−4.74	−4.79	−4.87	−4.83	−4.86	−4.88	−4.51	−5.00	−4.91	−4.90	−4.87	−4.77	−5.52	−4.60	−4.87	−4.78	−4.79	−5.52
FEXBOA_G		0	−4.30	−4.60	−4.69	−4.62	−4.53	−4.64	−4.48	−4.67	−4.65	−4.54	−5.02	−4.61	−4.57	−4.57	−4.57	−4.94	−4.00	−4.61	−4.42	−4.68	−5.02
		1	-	-	-	−4.45	-	-	-	-	−4.60	-	-	-	-	-	-	-	−5.35	-	-	−4.62	−5.35

## Data Availability

Data is available within the article and Supporting Information. An archived dataset including the docking results, geometry optimization results, CDGA Align output, and a README file has been deposited in Zenodo and is publicly available at https://doi.org/10.5281/zenodo.22730805 (accessed on 17 September 2026). The analyses in this study were performed using CDGA Align, which is described in a separate article published in Journal of Chemical Information and Modeling, available at: https://doi.org/10.1021/acs.jcim.6c02866 (accessed on 17 September 2026). The GitHub repository is publicly available at https://github.com/enapiorkowska/CDGA_Tool/ (accessed on 17 September 2026), and an archived version of the software used in this work is available at https://doi.org/10.5281/zenodo.21270534 (accessed on 17 September 2026).

## Supplementary Materials

The following supporting information can be downloaded at https://www.mdpi.com/article/10.3390/molecules31193379/s1, Table S1. List of 20 guest molecules used in this study, including their chemical structures, guest names derived from the corresponding refcodes and compound names, Table S2. GlideScore-based Top-N orientation recovery for each guest. Values of 1 and 0 indicate the presence and absence, respectively, of at least one Up pose within the specified Top N set. Table S3. Summary of guest-level Top-N recovery rates for Up poses, ranked by GlideScore, with 95% Confidence Intervals and comparisons to the 50:50 random-orientation baseline.
