# Supplementary material for: Can Molecular Docking Predict Guest Orientation in β-Cyclodextrin? A Cross-Docking and DFT Refinement Study
Source: Molecules. 2026 Sep 23;31(19):3379. doi: 10.3390/molecules31193379 (PMC13665827; doi:10.3390/molecules31193379)
Supplement: Supplementary file 1 [file molecules-31-03379-s001.zip › molecules-4560474-supplementary.pdf]

Table S1. List of 20 guest molecules used in this study, including their chemical structures, guest names derived from the corresponding refcodes and compound names.

Structure	Guest	Compound name
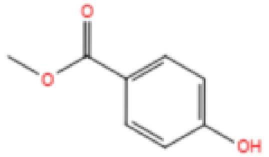	AJUVAC_G	methyl 4-hydroxybenzoate
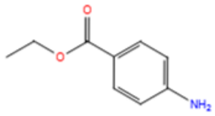	AVUDUS_G	ethyl 4-aminobenzoate
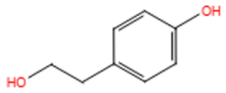	CIQFIU_G	4-(2-hydroxyethyl)phenol
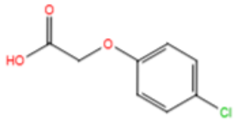	FEXBOA_G	(4-chlorophenoxy)acetic acid
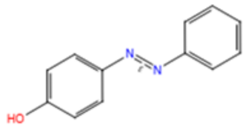	FODBEG_G	4-hydroxyazobenzene
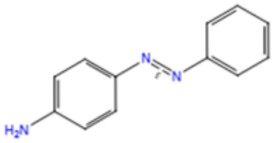	FODBIK_G	4-aminoazobenzene
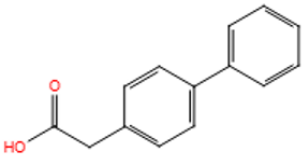	HAKPUG_G	biphenyl-4-ylacetic acid

Structure	Guest	Compound name
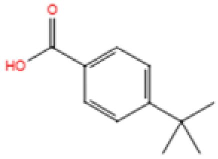	HEGXUM_G	4-t-butylbenzoic acid
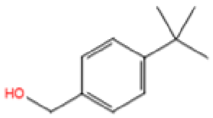	KOFJEU_G	4-t-butylbenzyl alcohol
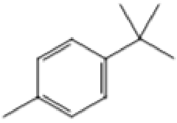	KUTJUE_G	4-t-butyltoluene
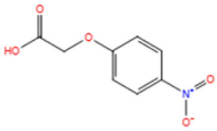	LACZEW_G	(4-nitrophenoxy)acetic acid
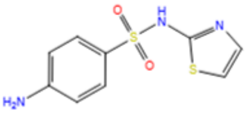	LILLUN_G	N-(2-thiazolyl)sulfanilamide
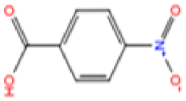	NILQUV_G	4-nitrobenzoic acid
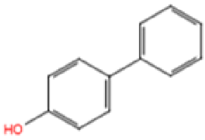	OFAXID_G	4-hydroxybiphenyl
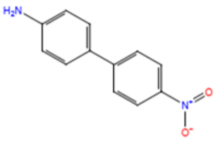	QACXEX_G	p-amino-p'-nitrobiphenyl

Structure	Guest	Compound name
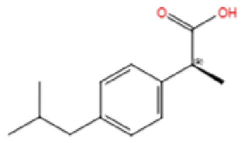	TUXKUS_G	S-(+)-ibuprofen
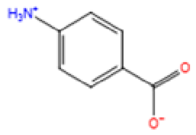	UPULEX_G	4-aminobenzoic acid
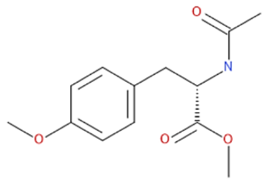	VOQDUA_G	N-acetyl-p-methoxy-L-phenylalanine methyl ester
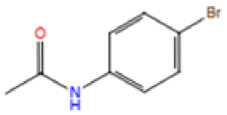	XADHIT_G	p-bromoacetanilide
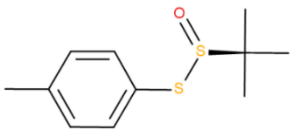	XUXFAY_G	S-p-tolyl t-butylthiosulfinate

Table S2. GlideScore-based Top-N orientation recovery for each guest. Values of 1 and 0 indicate the presence and absence, respectively, of at least one Up pose within the specified Top N set.

Guest	Top-1	Top-2	Top-3	Top-5
AJUVAC_G	1	1	1	1
AVUDUS_G	1	1	1	1
CIQFIU_G	1	1	1	1
FEXBOA_G	1	1	1	1
FODBEG_G	1	1	1	1
FODBIK_G	1	1	1	1
HAKPUG_G	0	1	1	1
HEGXUM_G	1	1	1	1
KOFJEU_G	0	1	1	1
KUTJUE_G	1	1	1	1
LACZEW_G	1	1	1	1
LILLUN_G	0	1	1	1
NILQUV_G	1	1	1	1
OFAXID_G	1	1	1	1
QACXEX_G	1	1	1	1
TUXKUS_G	1	1	1	1
UPULEX_G	0	1	1	1
VOQDUA_G	0	1	1	1
XADHIT_G	0	0	1	1
XUXFAY_G	0	1	1	1
**Average**	**65%**	**95%**	**100%**	**100%**

Table S3. Summary of guest-level Top-N recovery rates for Up poses, ranked by GlideScore, with 95% Confidence Intervals and comparisons to the 50:50 random-orientation baseline.

Metric	Observed	95% Wilson CI	50:50 baseline
Top-1	13/20 (65.0%)	43.3 - 81.9%	50.0%
Top-2	19/20 (95.0%)	76.4 - 99.1%	75.0%
Top-3	20/20 (100%)	83.9 - 100%	87.5%
Top-5	20/20 (100%)	83.9 - 100%	96.9%
